# Preferences for health outcomes associated with Group A Streptococcal disease and vaccination

**DOI:** 10.1186/1477-7525-8-28

**Published:** 2010-03-12

**Authors:** Grace M Lee, Joshua A Salomon, Charlene Gay, James K Hammitt

**Affiliations:** 1 Department of Population Medicine, Harvard Medical School and Harvard Pilgrim Health Care Institute, 133 Brookline Avenue, Boston, MA 02215, USA; 2 Division of Infectious Diseases, Department of Medicine, and Department of Laboratory Medicine, Children's Hospital Boston, 300 Longwood Avenue, Boston, MA 02115, USA; 3 Department of Global Health and Population, Harvard School of Public Health, 665 Huntington Avenue, Boston, MA 02115, USA; 4 Center for Risk Analysis and Department of Health Policy and Management, Harvard School of Public Health, 718 Huntington Avenue, Boston, MA 02115, USA

## Abstract

**Background:**

A 26-valent Group A Streptococcus (GAS) vaccine candidate has been developed that may provide protection against pharyngitis, invasive disease and rheumatic fever.  However, recommendations for the use of a new vaccine must be informed by a range of considerations, including parents' preferences for different relevant health outcomes. Our objectives were to: (1) describe parent preferences for GAS disease and vaccination using willingness-to-pay (WTP) and time trade-off (TTO) methods; and (2) understand how parents' implied WTP for a quality-adjusted life year (QALY) gained might vary depending on the particular health outcome considered (e.g. averted GAS disease vs. vaccine adverse events).

**Methods:**

Telephone interviews were conducted with parents of children diagnosed with GAS pharyngitis at 2 pediatric practice sites in the Boston metropolitan area. WTP and TTO (trading parental longevity for child's health) questions for 2 vaccine and 4 disease-associated health states were asked using a randomly selected opening bid, followed by a 2^nd ^bid and a final open-ended question about the amount willing to pay or trade. Descriptive analyses included medians and interquartile ranges for WTP and TTO estimates. The Wilcoxon signed-rank test was used to assess differences in WTP/QALY values for vaccine adverse events vs. disease states.

**Results:**

Of 119 respondents, 100 (84%) and 96 (81%) provided a complete set of responses for WTP and TTO questions, respectively. The median WTP and discounted (at 3% per year) TTO values to avoid each health state were as follows: local reaction, $30, 0.12 days; systemic reaction, $50, 0.22 days; impetigo, $75, 1.25 days; strep throat, $75, 2.5 days; septic arthritis, $1,000, 6.6 days; and toxic shock syndrome, $3,000, 31.0 days. The median WTP/QALY was significantly higher for vaccine adverse events (~$60,000/QALY) compared to disease states ($18,000 to $36,000/QALY).

**Conclusions:**

Parents strongly prefer to prevent GAS disease in children compared to vaccine adverse events. However, implied WTP/QALY ratios were higher for the prevention of vaccine adverse events. Regret for errors of commission vs. omission may differ and should be considered by vaccine policymakers.

## Background

Group A Streptococcus (GAS) is responsible for up to 2.6 million cases of pharyngitis in children each year and 1.1 million cases in adults in the U.S., with an estimated economic burden of $224-539 million annually due to GAS pharyngitis [1,2]. In addition, infection with GAS causes up to 9,700 cases of invasive disease and 1,300 deaths annually [3,4]. The clinical spectrum of invasive GAS disease is broad and may include bacteremia, pneumonia, septic arthritis, osteomyelitis, meningitis, necrotizing fasciitis, or streptococcal toxic shock syndrome [4-7].

Recently, a 26-valent GAS vaccine candidate was developed that may provide protection against pharyngitis, invasive disease and rheumatic fever [8,9]. Such a vaccine may reduce the burden of GAS disease by up to 85% in the U.S [4,10,11]. However, parental preferences regarding the prevention of disease vs. the risk for minor vaccine adverse events should be explicitly considered before recommending widespread use of a GAS vaccine. This is particularly relevant as concerns about vaccine safety have become prominent, and the number of parents refusing to vaccinate their children continues to grow as perceptions about the risks of vaccination may outweigh perceived benefits [12-15].

Estimating preferences for childhood vaccination programs has been challenging for several reasons. First, parents often serve as proxy respondents for young children, raising concerns about how to distinguish the child's well-being from the parent's altruism [16-18]. However, since parents are responsible for decision-making about vaccinating their own children, and young children are often unable to provide quantitative assessments of their preferences, the choice of parents as a proxy may be appropriate. Second, while many of the older childhood vaccines have been focused on preventing chronic disability and death (e.g. neurologic disability and death after *Haemophilus influenzae *type b infection, paralysis and death after polio infection), newer vaccines may target health conditions that are temporary in duration (e.g. otitis media and bacteremia in pneumococcal infection) [19,20]. Health states that are described as short-term may be valued differently than the same health states as chronic conditions [21-23]. Third, off-the-shelf utilities do not exist for many of the short-term health conditions, necessitating a formal assessment of preferences when implementing a new vaccination program. Finally, although many childhood vaccination programs have historically been found to be cost-saving, the higher prices associated with several newer vaccines and rising emphasis on concerns about vaccine safety require explicit examination of the costs and health consequences of new programs [12-15]. An empirical assessment of the willingness-to-pay for a quality-adjusted life year gained may provide important information for decision-makers in the context of national vaccine policy.

Thus, our objectives were to: (1) describe parent preferences for GAS disease and vaccination using willingness-to-pay (WTP) and time trade-off (TTO) methods, and (2) understand how parent's implied WTP for a quality-adjusted life year (QALY) gained might vary depending on the particular health outcome considered (e.g. averted GAS disease vs. vaccine adverse events).

## Methods

### Study Population

Telephone interviews were conducted with parents of children diagnosed with GAS pharyngitis at two pediatric practice sites in the Boston metropolitan area. We identified 236 potentially eligible episodes of GAS pharyngitis among children who were seen at one urban and one suburban practice for urgent care visits from October 1, 2005 to January 25, 2006. Families were considered eligible for the study if the child or adolescent was less than 18 years of age, had symptoms consistent with strep throat and a confirmed diagnosis of GAS pharyngitis with a positive rapid strep test or throat culture.

Fifty-two cases were excluded from the study for the following reasons: incorrect address or telephone number (22), child already had a sibling enrolled in the study (12), child previously enrolled in the study (7), taken to the doctor by someone other than a parent or guardian (7), parent or guardian did not understand that child had GAS pharyngitis (3), and parent or guardian was non-English speaking (2). Of the 236 potentially eligible children seen during the study period, 135 (57%) parents agreed to participate in the study. Among these parents, 16 were initially selected to pilot the preferences survey, and 119 parents received the final survey.

### Survey

The telephone survey included questions about parent preferences for avoiding short-term health states associated with GAS disease and vaccination in their child including impetigo, strep throat, septic arthritis, and streptococcal toxic shock syndrome (Please see Additional File 1). Parents were asked both willingness-to-pay (WTP) and time trade-off (TTO) questions about each health state. In addition, information was collected on demographics, duration of illness, medical costs, and non-medical costs associated with episodes of GAS pharyngitis in children.

For WTP questions, respondents were asked "Using money that is available to you today, think about how much money you would be willing to pay to prevent your children from having this condition." The framing of the question in terms of currently available resources was intended to elicit the amounts that individuals would actually pay in order to enhance the validity of the study; a disadvantage of this approach is that higher respondent incomes would likely be associated with higher WTP [24]. Interviewers presented respondents with a randomly selected opening bid (high, intermediate, low) for each health state (Please see additional File 2). If respondents were willing to pay the opening bid, they were asked if they would be willing to pay a higher amount to avoid the health state in question. If respondents were not willing to pay the opening bid, they were asked if they would be willing to pay a lower amount. After their response to the subsequent bid, interviewers asked, "What is the most you would be willing to pay?".

For TTO questions, respondents were instructed as follows: "Think about how many hours or days you would be willing to give up from the end of your life in order to prevent your child from having this condition." Of note, all health conditions were described as being short-term and the duration of each health state was described, ranging from 2 days to 3 weeks. Similar to WTP, respondents were given a high, intermediate, or low opening bid in terms of hours or days traded to prevent illness in their child. After answering, a subsequent bid was offered and then parents were asked, "What is the most you would be willing to trade?" Because parents were asked to trade time from the end of their lives, we estimated the impact of alternative assumptions about discounting (0-5%) of future health outcomes.

### Statistical analyses

Final responses to the binary plus follow-up questions for WTP (N = 100) and TTO (N = 96) are presented as medians and interquartile ranges (IQRs), in order to minimize the impact of outliers, particularly since the distributions of responses were skewed. Individuals who either refused to answer (N = 7 for WTP; N = 12 for TTO) or did not provide an open-ended response for each of the health states (N = 12 for WTP; N = 11 for TTO) were excluded from our primary analysis, in order to accommodate analysis of individual rankings of the set of health states.  In a secondary analysis, we also estimated predicted values for missing, interval or censored responses for each health state using a multivariable interval regression model that included age, gender, and income as covariates. Comparison of results including these predicted values allowed us to understand the impact of missing data on the primary analyses. To understand the impact of income on WTP estimates, we calculated Spearman's rank correlation coefficients.

Disutilities for short-term health states were calculated using a previously published method [21]. The numerator is the difference between the discounted stream of normal life expectancy (LE) for the respondent, in years, and the discounted stream of shortened LE, calculated as (1/r)*(1-e^(-r)*(LE of respondent)^) - (1/r)*(1-e^(-r)*(LE of respondent - time traded)^), where r is the discount rate. We interpret this expression as the number of QALYs given up by the parent to avoid having the child live with the health outcome under consideration.  The denominator is the duration of the health state for the child that begins at the present time, discounted accordingly for consistency, calculated as (1/r) * [1-e^(-r) (duration of health state)^]. We assumed that the maximum amount of discounted time traded from the end of the respondent's life could not exceed the (discounted) duration of the health state, which anchors the lowest time tradeoff value at zero. To calculate WTP for a QALY, we divided the WTP response by the same expression used in the numerator of the disutility calculation above, as this represents the QALY equivalent for the health outcome:

Individual values for the WTP/QALY ratio were calculated and medians and interquartile ranges are reported for each of the six health states described. Values for individuals who did not trade any time to avoid illness (i.e. denominator equals zero) were excluded for that particular health state for WTP/QALY calculations, although they were included in descriptive analyses for TTO responses. The Wilcoxon signed-rank test was used to test for differences in WTP/QALY values for each matched pair of health states. Additionally, 95% bias-corrected confidence intervals were calculated for the median WTP/QALY for each health state using bootstrapping with 10,000 re-samples. We chose to use the non-parametric bootstrapping method to derive our bias-corrected confidence intervals, since it makes no assumptions about the distribution of WTP/QALY value.

## Results

### Study population

Our study population included 119 parents of children diagnosed with GAS pharyngitis. Respondents were mostly female (91%), had at least some college education (80%), and had annual household incomes greater than or equal to $80,000 (50%). (Table 1) Among these participants, 100 (84%) and 96 (81%) individuals provided open-ended responses to the WTP and TTO questions, respectively, although 112 (94%) and 107 (90%) individuals provided at least some interval data for WTP and TTO questions. Comparing those who provided open-ended responses to those who did not, there were no differences in age, gender, educational level, or race/ethnicity. Respondents who refused to report their annual household income were significantly less likely to provide complete, open-ended responses to WTP (p = 0.002) and TTO (p = 0.001) survey items.

**Table 1:** Characteristics of respondents (N = 119)

	N (%)
Age of respondent (in quartiles) (N = 118)	
19-36 years	29 (24.6%)
37-40 years	32 (27.1%)
41-43 years	26 (22.0%)
44-62 years	31 (26.3%)

Female respondent (N = 119)	108 (90.8%)

Annual household income, 2005 US$ (N = 119)	
<50,000	26 (21.9%)
50,000-<80,000	22 (18.5%)
> = 80,000	60 (50.4%)
Refused to answer	11 (9.2%)

Educational level (N = 117)	
Up to high school graduate	22 (18.8%)
Some college/technical school	29 (24.8%)
College graduate	40 (34.2%)
Postgraduate	26 (22.2%)

Race/ethnicity (N = 117)	
White	91 (77.8%)
African American	16 (13.7%)
Hispanic	4 (3.4%)
Other	6 (5.1%)

Survey version (N = 119)	
Low opening bids	40 (33.6%)
Intermediate opening bids	41 (34.5%)
High opening bids	38 (31.9%)

### Willingness-to-pay

The median WTP values for local and systemic reactions associated with vaccination were $30 and $50, respectively (Table 2). Mild disease states associated with GAS infection such as impetigo and strep throat were associated with higher median WTP values. Parents were willing to pay the highest amounts to avoid severe disease such as septic arthritis ($1,000) or streptococcal toxic shock syndrome ($3,000). Nonetheless, some respondents reported they would not be willing to pay any amount of money ($0) to avoid the following health states: local reaction (12%), systemic reaction (6%), impetigo (4%), and strep throat (3%). Income was significantly correlated with WTP estimates for the most severe health states such as septic arthritis (ρ = 0.286, p = 0.003) and streptococcal toxic shock syndrome (ρ = 0.289, p = 0.003); however, there was no correlation between income and WTP values for local reaction, systemic reaction, impetigo, or strep throat. In secondary analyses that included predicted values for missing, interval, or censored data, the median WTP was the same or nearly the same for all states: local reactions ($30), systemic reactions ($50), impetigo ($75), strep throat ($85), septic arthritis ($1,000), and toxic shock syndrome ($3,233).

**Table 2:** Median open-ended WTP (N = 100) and TTO (N = 96) values for health states associated with Group A Streptococcal disease and vaccination.

Health state	Median WTP (25%-75%)	Median undiscounted days traded (25%-75%)	Median days traded discounted at 3% (25%-75%)	Median days traded discounted at 5% (25%-75%)
Local reaction	$30 (10-50)	0.42 (0.15-1.0)	0.12 (0.04-0.33)	0.05 (0.01-0.16)

Systemic reaction	$50 (20-50)	0.83 (0.19-1.83)	0.22 (0.05-0.43)	0.10 (0.02-0.19)

Impetigo	$75 (35-112.5)	1.25 (0.42-7.0)	0.41 (0.11-1.99)	0.21 (0.04-0.78)

Strep throat	$75 (30-150)	2.5 (0.50-10.0)	0.75 (0.15-3.61)	0.33 (0.07-1.83)

Septic arthritis	$1,000 (250-2,250)	21.0 (8.5-120.0)	6.56 (3.03-32.71)	2.98 (1.17-12.6)

Toxic shock syndrome	$3,000 (1,000-10,000)	90.0 (30.0-365.0)	31.0 (7.56-135.65)	14.2 (3.15-63.9)

### Time trade-off

We calculated the present value of the median number of days traded assuming discount rates of 0%, 3%, and 5% (Table 2). If we assumed that respondents discounted future time at 3% per year, the median number of days traded for a local reaction was 0.12 days compared to 31.0 days traded to avoid a case of toxic shock syndrome (Table 2). Of note, some respondents were unwilling to trade any time (zero days) for the following health states: local reaction (22%), systemic reaction (18%), impetigo (17%), strep throat (14%), septic arthritis (4%), and toxic shock syndrome (3%). For these short term health states, median utilities were calculated for local reactions (0.942), systemic reactions (0.892), impetigo (0.959), strep throat (0.925), septic arthritis (0.687), and toxic shock syndrome (0.0). Accounting for the duration of each health state (which ranged from 2 days to 3 weeks), median estimates for QALYs in the year of the infection were 0.9997 for local reactions, 0.9994 for systemic reactions, 0.9986 for impetigo, 0.9977 for strep throat, 0.9793 for septic arthritis, and 0.9063 for toxic shock syndrome. In a secondary analysis, the inclusion of predicted estimates for missing, interval or censored variables provided similar estimates for days traded discounted at 3%: local reactions (median 0.17; IQR [0.04-0.44]), systemic reactions (median 0.24; IQR [0.06-0.54]), impetigo (median 0.56; IQR [0.13-1.99]), strep throat (median 0.75; IQR [0.27-3.5]), septic arthritis (median 10.5; IQR [3.5-30.5]), and toxic shock syndrome (median 41.5; IQR [8.3-134]).

### WTP per QALY

**Figure 1:**
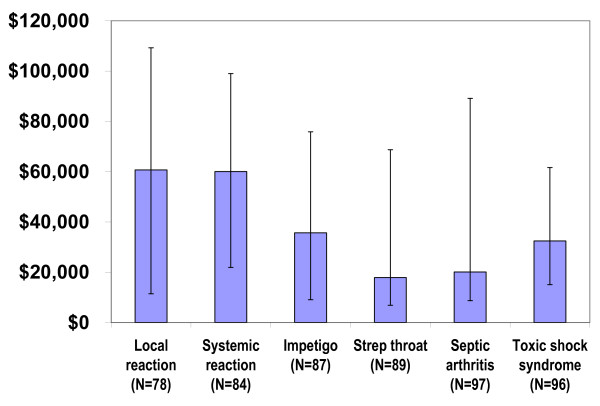
**Median (IQR) WTP per QALY**.

The median implied WTP per QALY and 95% confidence intervals were calculated across individuals for each health state (Figure 1). Local reactions had a significantly higher WTP per QALY when compared to strep throat (p = 0.006), septic arthritis (p = 0.029), or toxic shock syndrome (p = 0.034). The median WTP per QALY was also significantly higher for systemic reactions compared to any of the health states associated with GAS disease (impetigo, p = 0.012; strep throat, p = 0.033; septic arthritis, p = 0.008; toxic shock syndrome, p = 0.022). When we included WTP per QALY estimates based on predicted values, the median amount remained significantly higher for local reactions vs. septic arthritis (p = 0.019), local reactions vs. toxic shock syndrome (p = 0.044), systemic reactions vs. strep throat (p = 0.010), systemic reactions vs. septic arthritis (p = 0.008), and systemic reactions vs. toxic shock syndrome (p = 0.009).

## Discussion

Parents were willing to trade more time and money to avoid severe health states (i.e. septic arthritis, toxic shock syndrome) associated with GAS disease vs. mild GAS disease states (i.e. impetigo, strep throat) or minor vaccine adverse events. The relative strength of preference to avoid disease states, particularly severe conditions, compared to minor vaccine adverse events has been previously demonstrated [20,21]. Interestingly, however, many vaccine cost-effectiveness analyses have not typically considered the potential disutility associated with vaccine adverse events in program evaluations [19], perhaps because historically vaccines were life-saving, so these minor adverse events were negligible compared to the large overall benefits. As newer vaccines focus more on the prevention of morbidity, rather than mortality, parental and patient preferences to avoid both disease states and vaccine adverse events should be explicitly considered.

Prior studies have estimated parental WTP to avoid minor vaccine adverse events such as local or systemic reactions.  A 1999 study [20] reported a median parental WTP of $10 to $25 to reduce an infant's pain and emotional distress from childhood vaccination, while a study in 2001 reported a WTP of $25 to prevent fever and fussiness in young children after pneumococcal conjugate vaccine administration [25]. Another study conducted in 2002 examined parental WTP to avoid local and systemic reactions in adolescents, and found median estimates of $3 and $13, respectively [21]. Parents in our study reported slightly higher WTP values to prevent local ($30) and systemic ($50) reactions after a GAS vaccine, which may reflect differences in health state descriptions across studies, different considerations by parents depending on the age of the child (infant vs. toddler vs. adolescent), differences in the socioeconomic status of our population, inflation, or changes in the overall societal context regarding vaccine safety.

Empirically calculating the implied WTP per QALY may provide insight into the true societal WTP for gains in health, which may be preferred to using the persistent benchmark of $50,000 to $100,000 per QALY saved [26]. Although the standard approach in cost-effectiveness analysis relies on benchmarks for high-value interventions using the same threshold value for the WTP per QALY for all interventions, we observe substantial differences in the WTP per QALY to avoid different health states. In our study, parents were willing to pay more per incremental health gain to avoid vaccine adverse events (~$60,000 per QALY) compared to avoiding health states associated with GAS disease (~$18,000 to $36,000 per QALY). If these differences relate to true variability in the relative importance parents place on different types of outcomes, after controlling for the duration and severity of these outcomes, an important implication is that increased attention should focus on minimizing potential complications in healthy individuals. While it has previously been shown that treatment interventions are strongly preferred by society to preventive interventions [27,28], we are not aware of any studies that have explicitly compared preferences regarding vaccine adverse events vs. disease prevention.

Our findings that parents have a greater WTP per QALY for preventing vaccine adverse events compared to disease may be indicative of how individuals experience regret. An action, such as vaccinating a child, resulting in a potential adverse event may generate more regret than an inaction (i.e. refusing to vaccinate a child), even if a child becomes ill with a preventable disease [29-31]. This phenomenon is often characterized in terms of the distinction between "acts of commission" and "acts of omission", which is particularly relevant in the case of vaccination [32]. Parents may feel more guilt over agreeing to give a vaccine to their child that might cause harm, particularly in the short term, when compared to not vaccinating their child who by random chance develops disease. This may be reinforced by the changing perception of the risk-benefit balance by society, where fewer individuals have direct experience with vaccine-preventable diseases, furthering the intuitive response by some parents to focus more on vaccine safety and concerns about harming their child [33]. Further exploration of how regret for errors of commission and omission may influence parental preferences in vaccination programs is needed, particularly as new vaccines are recommended for use.

Our study has several limitations. First, our study population was relatively small and limited to parents of children who have experienced GAS pharyngitis. Consideration should be given to obtaining community values regarding GAS vaccination and disease [34]. Second, parents may not have had a complete understanding of the implications of these health states since our descriptions were brief and interviews were conducted by phone. As with any TTO, since parents were trading time from the end of their life, they may have assumed that they were trading time from a worse health state than their present condition and potentially have biased our TTO disutility estimates upward [35]. Third, anchoring bias may have occurred for our WTP and TTO estimates since we presented individuals with an initial opening bid that may have affected subsequent responses, although we did attempt to minimize this by randomizing among 3 different starting bids [36]. Fourth, missing or incomplete responses may have biased our WTP and TTO estimate in either direction. In a secondary analysis, however, our findings did not change significantly with the inclusion of predicted estimates for these individuals based on their characteristics. Fifth, WTP per QALY was inferred rather than directly elicited. Additionally, the pattern of declining WTP per QALY estimates for more severe health states may be due in part to the insensitivity to scale in WTP [37,38]. Finally, another key limitation of this study is that information was not available regarding parental refusal or deferral on any of their child's vaccines, thus we could not validate the WTP per QALY estimates with actual changes in behavior patterns.

Our findings suggest that parents prefer to prevent GAS disease in children compared to preventing minor vaccine adverse events, but that parents are also willing to pay more per QALY gained to prevent vaccine adverse events. Parental preferences should be incorporated in decision-making by policymakers when implementing new vaccination programs in the U.S.

## Competing interests

The authors declare that they have no competing interests.

## Authors' contributions

GL participated in the conception and design, acquisition of data, analysis and interpretation of data, drafting of the manuscript, statistical analysis, and the obtaining of funding. JS participated in the conception and design, analysis and interpretation of data, statistical analysis, and critical revision of the manuscript. CG participated in the acquisition of data, administrative, technical, and material support, and critical revision of the manuscript. JH participated in the conception and design, analysis and interpretation of data, and critical revision of the manuscript. All authors read and approved the final manuscript.

## Supplementary Material

Additional file 1 Description of health states. Click here for file

Additional file 2 Description of high, intermediate and low bid vectors used for WTP and TTO questions. Click here for file
