# Supplementary material for: Preferences for health outcomes associated with Group A Streptococcal disease and vaccination
Source: Health Qual Life Outcomes. 2010 Mar 12;8:28. doi: 10.1186/1477-7525-8-28 (PMC2848145; doi:10.1186/1477-7525-8-28)
Supplement: Additional file 1 — Description of health states. [file 1477-7525-8-28-S1.DOC]

Additional File 1. Description of health states

| Health state |  |
| --- | --- |
| Local reaction | Your child is perfectly well before vaccination. After receiving a vaccine, <he/she> has mild soreness of the upper arm and an area of redness that is about the size of a quarter lasting for 2 days. Your child is able to participate in usual activities without difficulty. |
| Systemic reaction | Your child is perfectly well before vaccination. After receiving a vaccine, <he/she> has slight fevers, body aches, and decreased energy that last for 2 days. Your child is able to participate in usual activities without difficulty. |
| Impetigo | Your child has a small patch of skin blisters that become crusted after several days. <He/she> goes to the doctor to get a prescription for an antibiotic ointment that is put on the blisters for 10 days. Although the blisters do not bother your child, <he/she> needs to miss 1 day of school to prevent spread to others. The skin blisters completely heal 5 days after starting treatment. |
| Strep throat | Your child has a sore throat that causes painful swallowing, slight fevers, and upset stomach. <He/she> goes to the doctor, gets a test for strep throat, and takes antibiotics by mouth for 10 days. With treatment, the illness lasts a total of 3 days and causes your child to miss 1 day of school to prevent spread to others. There is a 1 in 5 chance that the antibiotics will cause mild diarrhea. |
| Septic arthritis | Your child has an infection of the hip that causes fever, pain and limping. <He/she> goes to the doctor and is then sent to the Emergency Department and admitted to the hospital. A needle is put into the hip joint to diagnose the infection, and then <he/she> is taken to the operating room to clean the infected hip joint. Antibiotics are given through an IV tube in the arm. Your child stays in the hospital for 1 week and then is sent home to finish another 2 weeks of antibiotics by mouth. There is a 1 in 5 chance of getting mild diarrhea from antibiotics. After treatment, your child is completely healthy. |
| Toxic shock syndrome | Your child has high fevers, headaches, body aches, a sunburn-like rash and appears extremely sick. <He/she> is admitted to the hospital Intensive Care Unit for a breathing tube and for medicines through IV tubes in the arms and legs. <He/she> stays in the hospital for 3 weeks for treatment, but then recovers fully. |
