# Supplementary material for: Preferences for health outcomes associated with Group A Streptococcal disease and vaccination
Source: Health Qual Life Outcomes. 2010 Mar 12;8:28. doi: 10.1186/1477-7525-8-28 (PMC2848145; doi:10.1186/1477-7525-8-28)
Supplement: Additional file 2 — Description of high, intermediate and low bid vectors used for WTP and TTO questions. [file 1477-7525-8-28-S2.DOC]

Additional File 2. Description of high, intermediate and low bid vectors used for WTP and TTO questions.

|  | WTP  Initial bid [Range] | | | TTO  Initial bid [Range] | | |
| --- | --- | --- | --- | --- | --- | --- |
| Health state | High bid vector | Intermediate bid vector | Low bid vector | High bid vector | Intermediate bid vector | Low bid vector |
| Local reaction | $40  [30, 50] | $20  [10, 30] | $5  [2, 10] | 12 hrs  [6, 24] | 4 hrs  [2, 6] | 2 hrs  [1, 4] |
| Systemic reaction | $40  [30, 50] | $20  [10, 30] | $5  [2, 10] | 12 hrs  [6, 24] | 6 hrs  [2, 12] | 2 hrs  [1, 4] |
| Impetigo | $100  [50, 150] | $50  [25, 75] | $10  [5, 20] | 10 days  [5, 15] | 3 days  [1, 5] | 6 hrs  [2, 12] |
| Strep throat | $125  [50, 200] | $50  [25, 75] | $15  [5, 30] | 20 days  [10, 30] | 5 days  [2, 10] | 24 hrs  [6, 48] |
| Septic arthritis | $1000  [500, 2000] | $300  [100, 500] | $75  [50, 100] | 3 mo  [2, 4] | 30 days  [14, 60] | 7 days  [3, 14] |
| Toxic shock syndrome | $5000  [3000, 7000] | $2000  [1000, 3000] | $500  [100, 1000] | 6mo  [3, 12] | 60 days  [30, 90] | 14 days  [7, 30] |
